# Screening for Cognitive Impairment in African American Congregations

**DOI:** 10.1093/geroni/igaa057.2684

**Published:** 2020-12-16

**Authors:** Fayron Epps

**Affiliations:** Emory University, Atlanta, Georgia, United States

## Abstract

Having access to memory screenings is pivotal to early detection in the African American (AA) community. The purpose of this report is to describe the willingness and perceived barriers of AA congregants to participate in memory screenings. Out of 283 attendees to dementia-related church forums, 26% (n = 73) of the attendees participated in private memory screenings. The majority of the participants were female (88%, n = 64). Under half of the participants (37%, n = 27) scored below normal with 81% (n =22) being female. Several attendees declined the opportunity to have their memory screened for various reasons. These results support how women are disproportionately affected by cognitive impairment. Another alarming point was the low participation in memory screenings of event attendees. This report is important because it raises awareness of the need within the AA community, who are at a higher risk for memory loss, to receive screening. Part of a symposium sponsored by the Alzheimer’s Disease Research Interest Group.

